# MiR-146a negatively regulates dectin-1-induced inflammatory responses

**DOI:** 10.18632/oncotarget.16958

**Published:** 2017-04-08

**Authors:** Leilei Du, Xu Chen, Zhimin Duan, Caixia Liu, Rong Zeng, Qing Chen, Min Li

**Affiliations:** ^1^ From Institute of Dermatology, Jiangsu Key Laboratory of Molecular Biology for Skin Diseases and STIs, Chinese Academy of Medical Science and Peking Union Medical College, Nanjing 210042, China; ^2^ Jiangsu Province Blood Center, Nanjing, Jiangsu 210042, China

**Keywords:** MiR-146a, Candida albicans, β-glucan, dectin-1, inflammatory responses

## Abstract

Dectin-1 is the critical sensor for β-glucan from *Candida* which is the most common human fungal pathogen and cause superficial and system infection. MicroRNAs (miRNAs) play crucial roles in regulating innate immunity. However, the functional role of miRNAs in inflammatory response dependent on the activation of dectin-1 pathway has not been defined. In the present study, we found insoluble β-glucan from the cell wall of *Candida albicans* (*Ca*IG) was able to increase the production of of IL-6 and TNFα through Dectin-1-Syk-NF-κB and p38MAPK pathway. MiRNAs profiles combined with real-time PCR validation revealed that miR-146a, miR-30-5p, miR-210-3p expression level were increased in THP-1 cells treated with *Ca*IG. The interaction between Dectin-1 and *Ca*IG resulted in an long lasting increase of miR-146a expression dependent on Dectin-1-Syk-NF-κB, p38MAPK, contrasting with a rapid and transient increase of IL-6 and TNFα. Overexpression of miR-146a significantly suppressed the production of IL-6 and TNFα. MiR-146a mimics inhibited *Ca*IG-induced activity of p-IκBα and translocation of NF-κB p65. Luciferase reporter assays showed miR-146a inhibited NF-κB promoter-binding activity. Together, our data suggest miR-146a may play the potent negative feedback regulator in inflammatory response following Dectin-1 stimulation.

## INTRODUCTION

*Candida* species are most common cause of opportunistic fungal pathogens infections. As a result of the growing number of immunosuppressive population over the recent decade, the incidence of life-threatening invasive *Candida* infections has dramatically increased. *C. albicans* is the major species responsible for superficial and system candidiasis [1–4].

Innate immune system including neutrophils, monocytes, macrophages and dendritic cells constitute the first line of host defense against *Candida* infection. *Candida* cell walls are mainly composed of β-glucan, mannan and chitin. Recognition of these polysaccrides by pattern-recognition receptors (PRRs) leads to the activation of innate immune responses. Toll-like receptors (TLRs) TLR2 and 4, C-type lectin receptors (CLRs) including dectin-1, dectin-2, mannose receptor (MR), DC-SIGN, Galectin-3 and Mincle are sensors for *Candida* invasion [5–14]. Dectin-1 as the key β-glucan receptor has been demonstrated to induce phagocytosis, the respiratory burst and the production of cytokines and chemokines. The interaction between Dectin-1 and β-glucan can trigger two intracellular signaling transduction pathways, the spleen tyrosine kinase (Syk)-caspase recruitment domain-containing protein 9 (CARD9) pathway and the RAF pathway [15–18].

MicroRNAs (miRNAs) are a class of small noncoding RNAs that regulate gene expression at the post-transcriptional level via the RNA interference mechanism [19]. MiRNAs play crucial roles in regulation of immune and inflammatory response [20]. Previous studies have shown that miR-155, miR-146a, miR-146b, miR-125a and miR-455 can be up-regulated by heat killed *C. albicans* in Bone marrow derived macrophages [21]. MiR-146 and miR-155 have been reported to negatively regulate the production of inflammatory cytokines upon TLRs activation [22, 23]. However, whether miRNAs are regulated to suppress inflammatory responses after dectin-1 stimulation remains unknown. Hence, a microarray analysis was initially conducted to identify alterations in the miRNAome in THP-1 cells after dectin-1 activation trigger with *Ca*IG. Subsequent experiments were conducted to investigate the regulation role of miR-146a in inflammatory response dependent on the activation of dectin-1 pathway and related mechanism.

## RESULTS

### *Ca*IG triggers the expression and secretion of IL-6 and TNFα through Dectin-1-Syk pathway

**Figure 1:**
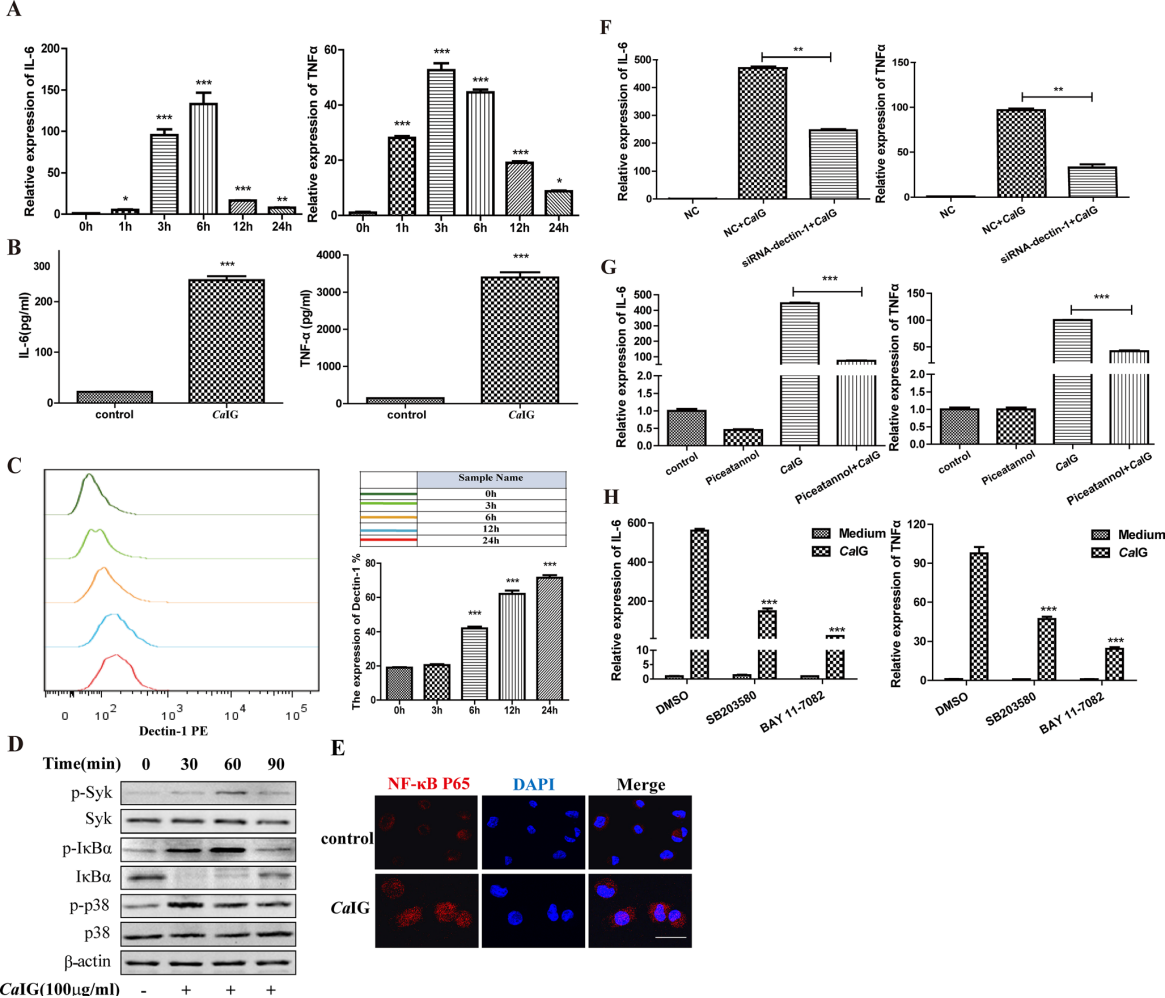
*Ca*IG induces the transcription and expression of IL-6, TNFα involving the dectin-1-Syk pathways (**A**) RNA was harvested at 0, 1, 3, 6, 24 hours after treated with 100 μg/ml *Ca*IG and subjected to gene expression analysis by qRT–PCR normalized to β-actin expression. For both genes analyzed, the significant difference of the *Ca*IG treated cells was compared with the corresponding untreated cells. (**B**) The culture medium of THP-1 cells treated with 100 μg/ml *Ca*IG for 24 hours was analyzed by ELISA to determine the protein level of IL-6 and TNFα. (**C**) The expression of dectin-1 in THP-1 cells. The THP-1 cells (2 × 10^5^ cells) were incubated with anti-human primate Dectin-1 monoclonal antibody for flow cytometric analysis. (**D**) Representative images of Western Blot analyses of p-Syk, Syk, p-IκBα, IκBα, p-p38 and p38 protein levels. Protein extracts were made from untreated cells and from *Ca*IG treated cells for 30, 60 and 90 minutes. Extracted protein samples (50 μg per lane) were subjected to electrophoresis and immunoblotting with antibodies specific for the 6 proteins and β-actin as control for equal loading. (**E**) NF-κB p65 translocation was analyzed by staining with NF-κB-p65 (red); and nucleuses were colored with DAPI (blue). Scale bar = 20 μm. (**F**) THP-1 cells were transfected with dectin-1 specific siRNA (siRNA-dectin-1) or scrambled control siRNA (NC) for 48 hours, and were subsequently treated with *Ca*IG for 6 hours. Total RNA was collected and IL-6 and TNFα expression levels were determined by qRT–PCR and normalized to β-actin expression. (**G**) THP-1 cells were treated with the inhibitor of Syk (piceatannol), (**H**) NF-κB (BAY 11-7082) or p38MAPK (SB203580) and were exposed to *Ca*IG 30 min later for 6 hours. Total RNA was collected and IL-6 and TNFα expression levels were determined by qRT–PCR and normalized to β-actin expression. Values are means ±S.E.M. from three experiments performed in triplicate. *P < 0.05; **P < 0.01; ***P < 0.001.

The time course study mRNA of expression of IL-6 and TNFα in THP-1 cells exposed to 100 μg/ml *Ca*IG was detected using real-time PCR. *Ca*IG induced the expression of IL-6 and TNFα in a time-dependent manner. *Ca*IG significantly up-regulated the mRNA expression of IL-6 and TNFα starting at 1 hour after stimulation, reached a peak at 4 hour and 6 hour respectively, and maintained the elevated level at 24 hour post stimulation (Figure 1A). The concentration of IL-6 and TNFα in the culture media of THP-1 cells after exposure to *Ca*IG (100 μg/ml) 24 hours was measured by ELISA. The secretion of IL-6 and TNFα was up-regulated by *Ca*IG (Figure 1B). Flowcytometry was used to analysis whether *Ca*IG could regulate the protein expression of Dectin-1 in THP-1 cells. The results showed that exposure to *Ca*IG highly up-regulated the expression of Dectin-1 (Figure 1C). To determine the activation of spleen tyrosine kinase (Syk), phosphorylation of Syk in THP-1 clles was assessed by western blotting. THP-1 clles challenged with *Ca*IG (100 μg/ml) for 30 mins showed the significant Syk phosphorylation was induced (Figure 1D). To explore the effect of *Ca*IG on activation of NF-κB in THP-1 cells, the phosphorylation and degradation of IκB-α was assessed by western blotting and nuclear translocation of NF-κB p65 observed using confocal microscopy. The results showed there was apparent IκB-α phosphorylation, degradation and nuclear translocation of NF-κB p65 in THP-1 cells stimulated with *Ca*IG (Figure 1D, 1E). Western blotting result showed the phosphorylation p38MAPK was increased in THP-1 cells the treatment of *Ca*IG (Figure 1D).

To determined whether Dectin-1 was involved in the producation of proinflammatory cytokine challenged with *Ca*IG, silencing of Dectin-1 by specific small interfering RNA (siRNA; Supplementary Figure 1A, 1B) abolished significantly the induction of IL-6 and TNFα (Figure 1F). Syk inhibitor (Piceatannol), NF-κB inhibitor (BAY 11-7082) and p38MAPK inhibitor (SB203580) attenuated the enhanced expression of IL-6 and TNFα induced by *Ca*IG (Figure 1G, 1H). These data strongly suggest that *Ca*IG is recognized by dectin-1, and then activates Syk-dependent signaling pathway, results in activation of NF-κB and p38MAPK, and induction of proinflammatory cytokines, including tumor necrosis factor α (TNF-α) and interleukin 6 (IL-6).

### Profiling miRNA expression of THP-1 cells induced by the interaction between Dectin-1 and *Ca*IG and qRT-PCR validation

**Figure 2:**
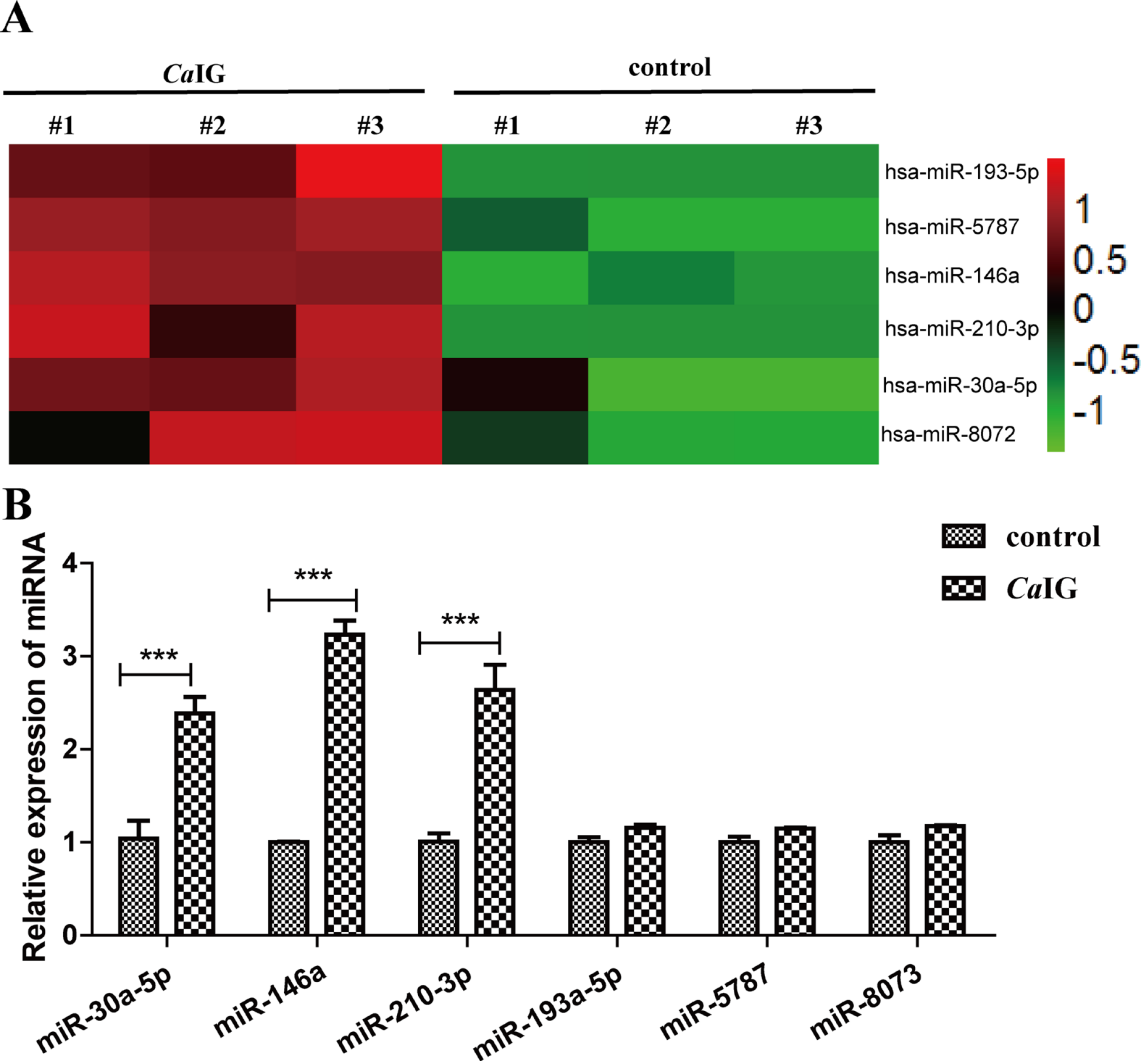
Characteristic alteration in the miRNAome of *Ca*IG treated THP-1 cells (**A**) Aberrantly expressed miRNAs of THP-1 cells after treated with 100 μg/ml *Ca*IG are displayed as a heat map, green indicating low expression, red indicating high expression. (**B**) The aberrantly miRNAs were analyzed using qRT-PCR normalized to U6 expression. Values are means ± S.E.M. from three experiments performed in triplicate.. ***P < 0.001.

To identify alterations in the miRNAome in THP-1 cells after dectin-1 activation trigger with *Ca*IG, a microarray analysis was conducted on total RNA from THP-1 cells stimulated by 100 μg/ml *Ca*IG for 24 hours (*n* = 3) and unstimulated controls (*n* = 3). 6 miRNAs which significantly increased (> two fold changes, *P* < 0.01) were identified (Figure 2A). No miRNAs were found to be significantly decreased in *Ca*IG stimulated compared to non-stimulated THP-1 cells. We further validated all the increased miRNAs using real-time quantitative reverse transcription-PCR (qRT-PCR). Slightly different with the microarray data, only three miRNAs (miR-146a, miR-30a-5p, miR-210-3p) are significantly increased (*P* < 0.001) in THP-1 cells after *Ca*IG treated (Figure 2B).

### MiR-146a is induced upon engagement of Dectin-1 in THP-1 cells

**Figure 3:**
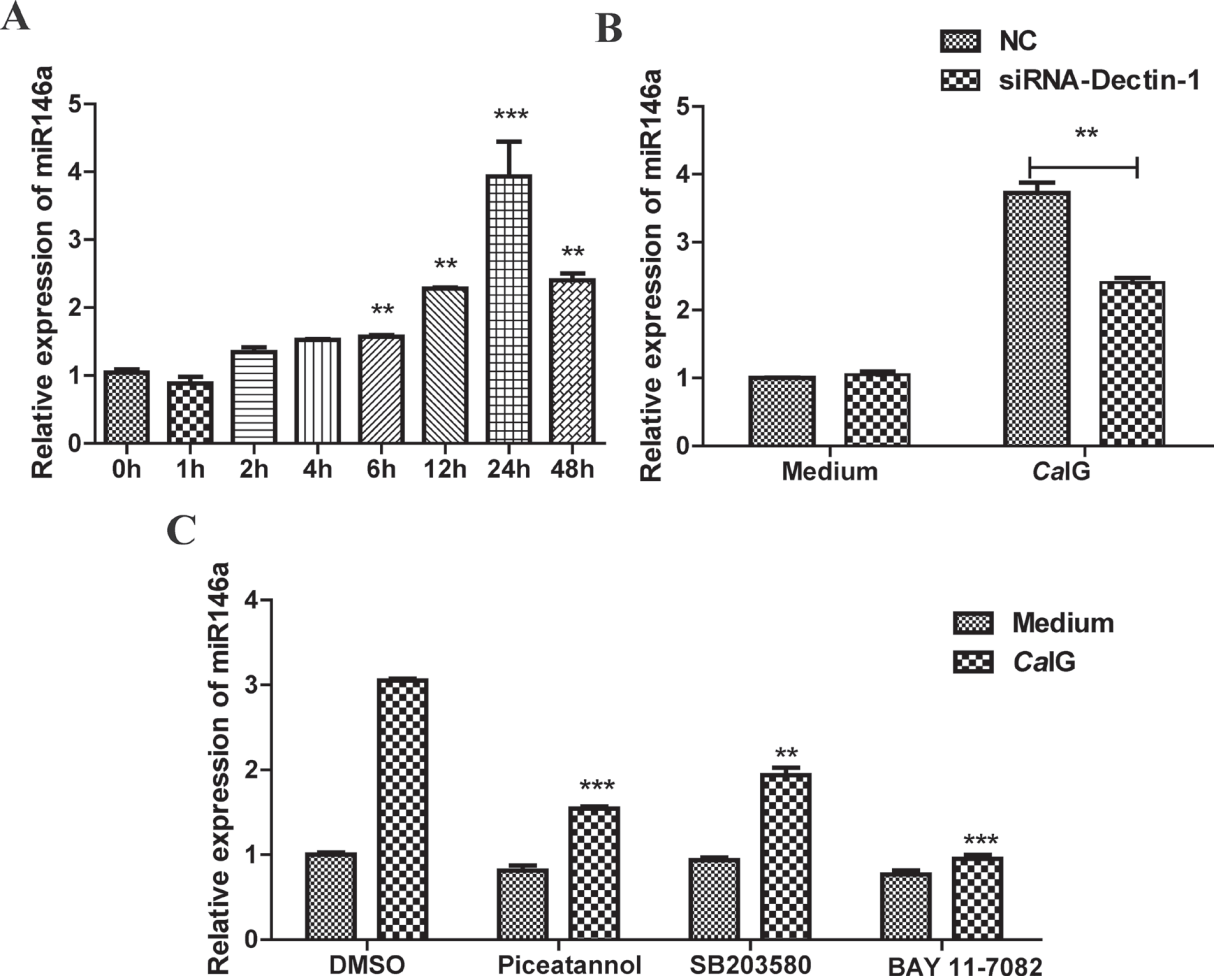
*Ca*IG induces miR-146a, involving the Dectin-1, NF-κB, and p38 MAPK pathways (**A**) THP-1 cells were treated with 100 μg/ml *Ca*IG. Total RNA was collected 0–48 hours later and miR-146a expression level was determined by qRT–PCR. (**B**) THP-1 cells were transfected with Dectin-1-specific siRNA (siRNA-Dectin-1) and subsequently treated with 100 μg/ml *Ca*IG. miR-146a expression was measured after 24 hours by qRT–PCR. (**C**) THP-1 cells were treated with chemical inhibitors for NF-κB (BAY 11-7082) p38MAPK (SB203580) and Syk (Piceatannol) were exposed to *Ca*IG 0.5 hour later. Relative RNA levels of MiR-146a were analyzed using qRT-PCR normalized to U6 expression. Values are means ± S.E.M. from three experiments performed in triplicate. **P < 0.01; ***P < 0.001.

In this study, we found *Ca*IG induced the expression of miR-146a in a time-dependent manner. Exposure to 100 μg/ml *Ca*IG resulted in significant expression of miR-146a. MiR-146a was strongly upregulated by *Ca*IG at 6 hours, the response peaked at 24 hours and remained significantly elevated at all time points studied, up to 48 hours (Figure 3A). Next studies were undertaken to characterize the expression of miR-146a in THP-1 cells upon Dectin-1 stimulation. Exposure to the Dectin-1 ligand *Ca*IG induced miR-146a expression in THP-1 cells after 24 hours. Silencing of Dectin-1 by specific small interfering RNA (siRNA-Dectin-1) abolished significantly the induction of miR-146a by *Ca*IG (*P* < 0.01; Figure 3B), demonstrating that induction of miR-146a by *Ca*IG was Dectin-1 dependent.

To explore the intracellular pathways that lead to the induction of miR-146a upon Dectin-1 stimulation, we used specific chemical inhibitors that target pathways known to act downstream of Dectin-1 signaling, i.e., the Syk, NF-κB and p38MAPK pathways. We used inhibitors for Syk (Piceatannol), NF-κB (BAY 11-7082) and p38MAPK (SB203580). None of these inhibitors had an effect on the miR-146a baseline expression (Figure 3C), suggesting that these pathways are not involved in the baseline transcription of the miR-146a gene. Induction of miR-146a by *Ca*IG was reduced by pretreatment with the Syk inhibitor piceatannol or NF-κB inhibitor BAY 11-7082 and p38MAPK inhibitor SB203580 (Figure 3C). These results demonstrate that *Ca*IG-induced miR-146a expression in THP-1 cells is mediated via the Syk-NFκB pathway and Syk-p38MAPK pathway.

### MiR-146a suppresses the *Ca*IG-induced production of inflammatory mediators in THP-1 cells

**Figure 4:**
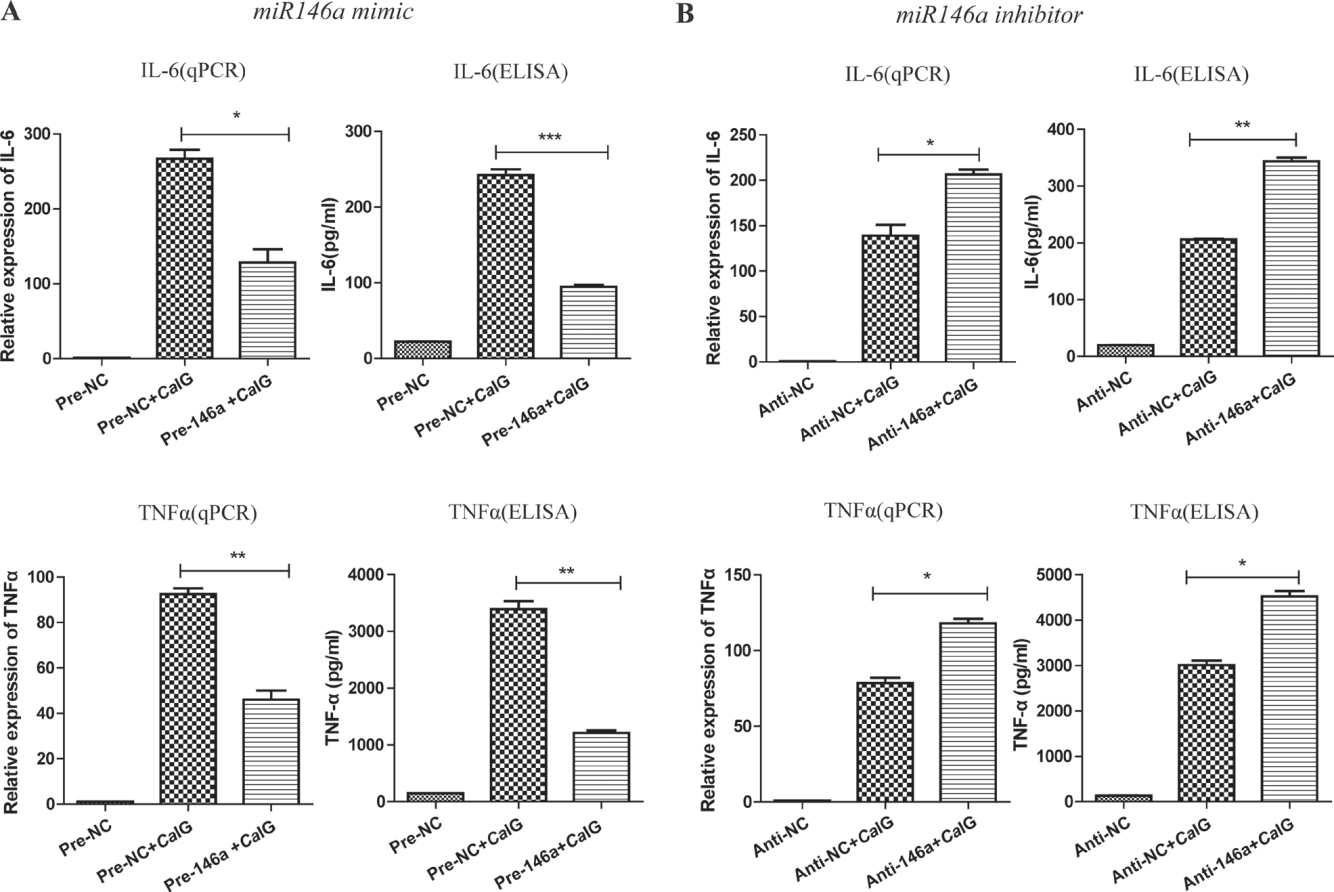
MiR-146a suppresses the *Ca*IG-induced production of IL-6 and TNF-α in THP-1 cells THP-1 cells were transfected with (**A**) miR-146a precursor (Pre-146a) or scrambled control precursor (Pre-NC); (**B**) miR-146a inhibitor (Anti-146a) or scrambled control inhibitor (Anti-NC). After 48 hours, cells were exposed to 100μg/ml *Ca*IG. The expression and secretion of IL-6 and TNF-α were determined 6 and 24 hours later using qRT–PCR and ELISA, respectively. Values are means ± S.E.M. from three experiments performed in triplicate. *P < 0.05; **P < 0.01; ***P < 0.001.

To assess the functional relevance of miR-146a expression under inflammatory conditions, we studied the effect of miR-146a on Dectin-1-induced expression of selected inflammatory mediators. To that end, we transfected THP-1 cells with synthetic miR-146a or scrambled control oligonucleotides (Supplementary Figure 1C). THP-1 cells were stimulated with *Ca*IG 48 hours after transfection and the expression and secretion of the inflammatory mediators IL-6 and TNF-α were determined 6 and 24 hours later via quantitative real-time reverse-transcriptase-PCR and ELISA. Overexpression of miR-146a markedly suppressed Dectin-1-induced expression and production of IL-6 and TNF-α (Figure 4A). To determine whether the Dectin-1-induced endogenous miR-146a can act as a negative feedback in THP-1 cells, we inhibited miR-146a expression using specific inhibitors or scrambled controls before *Ca*IG treatment (Figure 4B). Inhibition of endogenous miR-146a further increased Dectin-1-induced expression and production of IL-6 and TNF-α, demonstrating that *Ca*IG induced miR-146a expression acts as a negative feedback on this pathway.

### MiR-146a regulates *Ca*IG-induced inflammatory response of THP-1 cells through NF-κB signaling pathway

**Figure 5:**
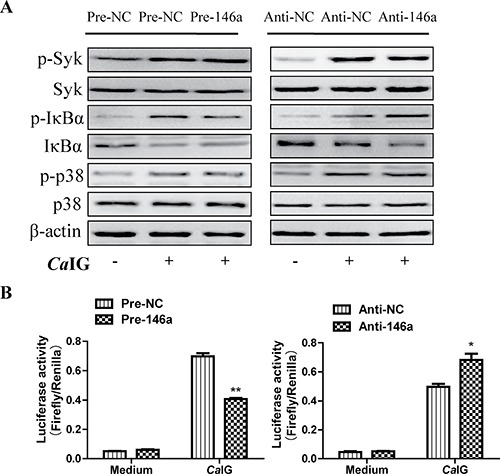
MiR-146a regulates *Ca*IG-induced inflammatory response of THP-1 cells through NF-κB signaling pathway (**A**) THP-1 cells were transfected with miR-146a precursor (Pre-146a) or scrambled control precursor (Pre-NC); miR-146a inhibitor (Anti-146a) or scrambled control inhibitor (Anti-NC). After 48 hours, cells were exposed to 100 μg/ml *Ca*IG. THP-1 cells lysates were collected 0.5 and 1 hours after exposed to *Ca*IG. Western Blot analysis of p-Syk, Syk, p-IκBα, IκBα(1 hours after exposed to *Ca*IG), p-p38 and p38(0.5 hours after exposed to *Ca*IG) protein levels using appropriate antibodies and β-actin as control for equal loading. (**B**) THP-1 cells were co-transfected with an NF-κB luciferase reporter plasmid and miR-146a precursor/inhibitor or regarding controls for 48 hours, exposed to medium or 100 μg/ml *Ca*IG, and luciferase activity was measured after 6 hours. Values are means ± S.E.M. from three experiments performed in triplicate. *P < 0.05; **P < 0.01.

**Figure 6:**
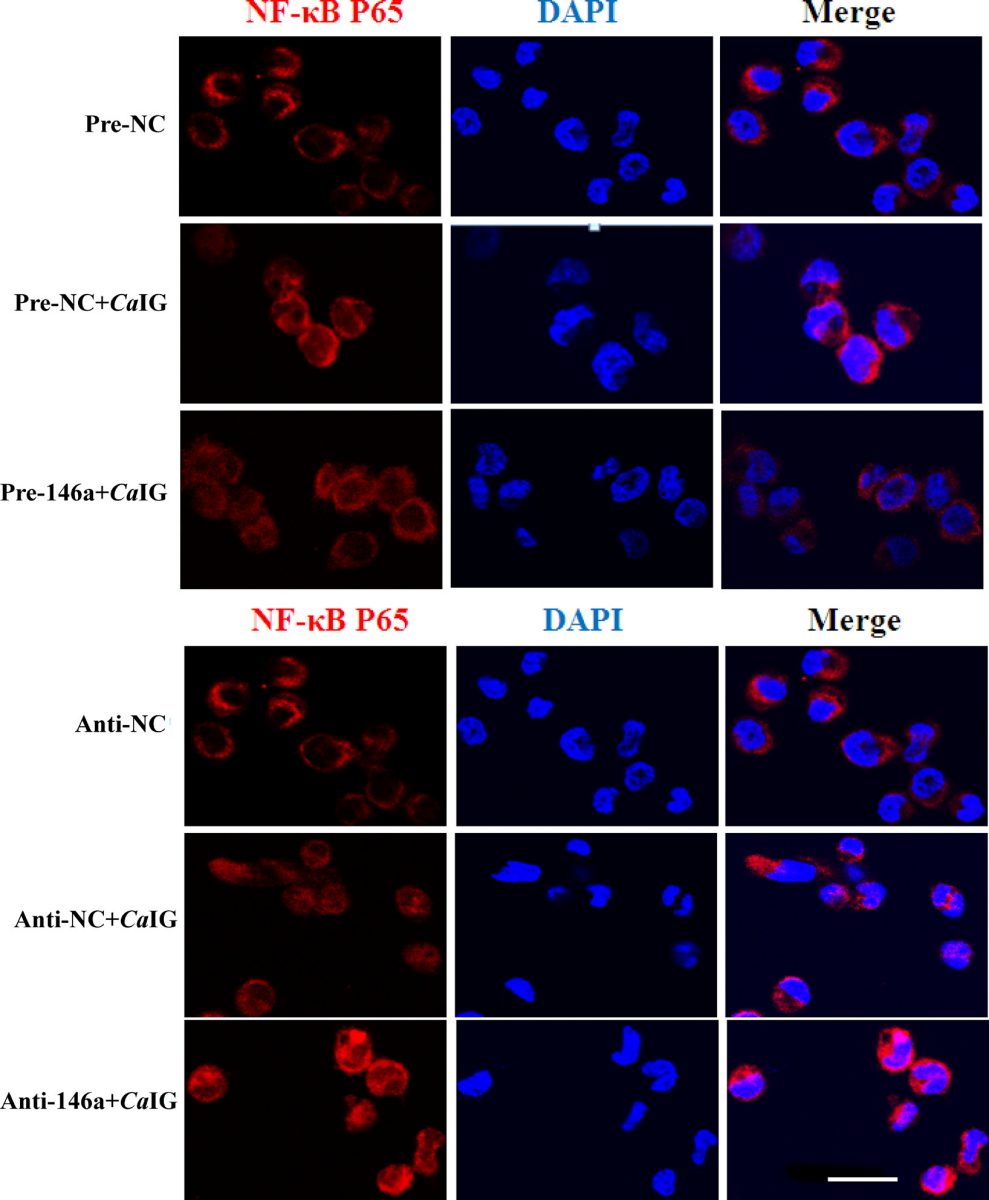
MiR-146a suppresses the *Ca*IG -induced translocation of NF-κB P65 in THP-1 cells THP-1 cells were transfected with miR-146a precursor (Pre-146a) or scrambled control precursor (Pre-NC); miR-146a inhibitor (Anti-146a) or scrambled control inhibitor (Anti-NC). After 48 hours, cells were exposed to 100 μg/ml *Ca*IG for 1 hours. NF-κB p65 translocation was analyzed by staining with NF-κB-p65 (red); and nucleuses were colored with DAPI (blue). Scale bar = 20 μm.

To determine whether suppression of inflammatory mediators via miR-146a was dependent on Syk, NF-κB and p38MAPK signaling molecules. In our study, we found that in THP-1 cells, overexpression and knock down of miR-146a had no influence to the *Ca*IG-induced activity of Syk and p38MAPK pathways. While the *Ca*IG-induced p-IκBα level was suppressed after the over-expression of miR-146a (Figure 5). Conversely, inhibition of endogenous miR-146a upregulated the *Ca*IG-induced activity of p-IκBα (Figure 5). In addition to these, the *Ca*IG-induced translocation of NF-κB p65 was also blocked due to the over-expression of miR-146a (Figure 6). We further transfected an NF-κB luciferase reporter into THP-1 cells. After *Ca*IG stimulation, the results of measurement of luciferase activity showed there was a 15-fold increase in the NF-κB-dependent DNA-binding activity. *Ca*IG-induced NF-κB activity was significantly suppressed after the over-expression of miR-146a. Inhibition of endogenous miR-146a further increased *Ca*IG-induced NF-κB activity (Figure 5A). Altogether, these data imply that MiR-146a regulates *Ca*IG-induced inflammatory response of THP-1 cells through NF-κB Signaling.

## DISCUSSION

Monocytes and monocyte-derived macrophages take part in host innate immune responses against *Candida* through the interaction between expressed PRPs and the *Candida* cell wall polysaccharide components. Our previous report showed that *Ca*IG, the highly branched polymers of D-glucose containing β-(1,3) and β-(1,6) linkage isolated from *C. albicans* cell wall, induced the production of cytokine and chemokine (TNF-α and IL-8) and H_2_O_2_ release [24]. Herein, we further demonstrated that *Ca*IG as a dectin-1 ligand was able to trigger activation of dectin-1 downstream signal molecules including Syk, NF-κB and p38MAPK, upregulate secretion of IL-6, TNF-α in the dectin-1-Syk-NF-κB and p38MAPK signaling pathway dependent manner.

MiRNAs have also been shown to play crucial roles during infection by diverse pathogens, including viruses, parasites and bacteria [25]. The activation of PRRs including Toll-like receptors (TLRs), C-type lectin receptors (CLRs), cytosolic proteins such as NOD-like receptors (NLRs) and RIG-I-like receptors (RLRs) which is vital for innate immune defense against microbial pathogens [26]. It is becoming increasingly evident that miRNA execute important regulatory functions in TLR signaling. In this study, we first explored whether miRNAs have roles in Dectin-1 signaling. Our study reveals the dectin-1 ligand *Ca*IG induced significant up-regulation of and no down-regulation using combination microarray analysis with real-time PCR validation. TLR2, TLR4, TLR7 and TLR9 play roles in the initiation of immune responses against *Candida* albicans [27]. Profiles of miRNA expression upon TLRs activation have been described. There are upregulation of miR-155, miR-146, miR-132, miR-147, miR-9, miR-21, miR-223, miR-125b, miR-27b, let-7e and down-regulation of miR-125, let-7i, miR-98 following TLR4 stimulation [22, 23, 28–35]. It has been showed the expression of miR-155, miR-146, miR-147, miR-9 increased through TLR2-dependent induction [22, 23, 28]. The up-regulated expression of miR-155 and miR-132 by TLR9 activation [22, 28], miR-9 by TLR7 activation [28], respectively. In comparison to above data, our observations suggest that only miR-146a is the common to both dectin-1 activation and TLR2, 4 signaling.

Monk *et al*. reported that miR-455, miR-125, miR-146 and miR-155 were up-regulated in murine bone marrow derived macrophages (BMDMs) stimulated with heat killed C. albicans [21]. In agreement with their results, we also found miR-146a increased in THP-1 cells treated with dectin-1 ligand *Ca*IG. However, in contrast to their results, the up-regulation of miR-455, miR-125 and miR-155 was not be detected in our study. The reasons of the differences between our results and their results are unclear but may be related to the different cells used *in vitro* study (human THP-1 monocytes in our study vs murine bone marrow derived macrophages in their study), different stimulator (insoluble β-glucan from the cell wall of *C. albicans* in our study vs heat killed *C. albicans* in their study).

Since miR-146a has been shown to act as a crucial negative regulator in inflammation and innate immune response by emerging evidence [36], we were particularly attracted to miR-146a from dectin-1 ligand *Ca*IG- induced miRNAs. Taganov [22] *et al*. firstly demonstrated that TLR4 ligand LPS and TLR2 agonist (peptidoglycan and its synthetic analog, Pam3CSK4) increased miR-146a expression in THP-1 cells. Similar to miR-146a induction by TLR2, 4 ligands, *Ca*IG could up-regulate the expression of miR-146a in THP-1 cells dependent on dectin-1, which further confirmed by abolishment of miR-146a level through silencing of dectin-1. Thus, it is possible that miR-146a is a common element of the host innate immune response. Dectin-1-induced inflammatory response is known to trigger special intracellular signal transduction pathways involving activation of Syk, NF-κB and p38MAPK [17, 27]. To investigate whether miR-146a up-regulation is regulated via the dectin-1 signaling pathway, we compared miRNA level between pretreatment with and without Syk, NF-κB and p38MAPK inhibitors. Our results provide the first evidence that the regulation of dectin-1 dependent miR-146a expression may relate to the activation of Syk, NF-κB and p38MAPK pathway. Moreover, our data showed miR-146a expression did not return to baseline level but remained significantly elevated up to 48 hours in contrast to the transient induction of proinflamatory cytokines (TNFα and IL-6) following the challenged with *Ca*IG. The observed kinetics of miR-146a expression upon Dectin-1 stimulation suggested that miR-146a may be involved in the resolution of the inflammatory response.

It is reported that over expression of miR-146a could inhibit TNFα production in THP-1 cells induced by LPS [37]. Moreover, miR-146a knock-out mice treated with LPS could develop an exaggerated pro-inflammatory response [38]. In this study, we investigated miR-146a function by over-expression and inhibition and showed that miR-146a negatively regulated pro-inflammatory cytokines (TNFα and IL-6). Our findings demonstrate miR-146 may “fine-tune” the innate immune response triggered by dectin-1, prevent an uncontrolled inflammatory response and return to homeostasis after *candida* infection.

TLR signaling molecules including MYD88, mAL, IRAK1, IRAK2, TRAF6, BTK and TAB2 have been shown to be direct targets of miRNAs [39]. Taganov *et al*. found that miR-146a could decrease the protein level of IRAK1, TRAF6 through interact with their mRNA in the TLR signaling pathway, which suppresses the secretion of inflammatory cytokines [22]. Hou *et al*. reported that miR-146a decreased the production of type-I interferon by targeting not only IRAK2 and TRAF6, but also IRAK1 [40]. During inflammatory response, miR-146a controlled the amplitude of the Ly-6Chi monocyte response through combining with Relb which the member of the noncanonical NF-κB/Rel family and identified as a direct miR-146a target [41]. It has been showed that miR-146a which expressed in regulatory T cells could suppress Th1 responses by targeting STAT1 [42]. In the present study, we showed that miR-146a inhibited dectin-1 downstream signaling NF-κB activation and negatively regulated proinflamatory cytokines (TNFα and IL-6) expression. Future studies will be needed to reveal the global expression of targeting molecules in the dectin-1 signaling pathway regulated by miR-146a and the role of the role of miR-146a in modulating systemic *Candida* infection with *in vivo* experiment models.

In summary, the present study indicates that dectin-1-ligand (*Ca*IG)-induced miR-146a acts as a negative feedback regulator of inflammation through down-regulation of the NF-κB pathway. Thus, miR-146a may contribute to the resolution of inflammation by shutting down excess inflammatory responses against *candida* infection. Investigation of the molecular regulation mechanisms of miR-146a in the inflammatory consequences triggered by *Candida albicans* may provide better understanding of the pathogenesis of candidiasis and useful information for developing potential therapeutic interventions against the disease.

## MATERIALS AND METHODS

### Cell lines and culture conditions

Human monocyte leukemia THP-1 cells were provided by American Type Culture Collection (ATCC). The cells were cultured in RPMI-1640 medium (Gibco, Grand Island, NY, USA) supplemented with 10% fetal bovine serum (FBS) (Gibco), and were maintained at 37°C in a humidified incubator under an atmosphere of 95% air and 5% CO_2_. For the preparation of differentiated THP-1 macrophage, THP-1 cells were incubated in a culture plate with medium including FBS and 100 ng/ml phorbol 12-myristate-13-acetate (PMA) (Sigma, St. Louis, MO, USA) for 48 h. At the end of the incubation, the cells were washed with PBS three times to exclude undifferentiated THP-1 cells, and then were replaced into medium without PMA, which was used throughout this study. The insoluble β-glucan from the cell wall of *Candida albicans* (*Ca*IG) was isolated according to the method described elsewhere [24]. For inhibiting signaling pathways, THP-1 cells were pretreated with piceatannol (Sigma, St. Louis, MO, USA) at 50 μM concentration or BAY-11-7082 (Sigma, St. Louis, MO, USA), SB203580 (Sigma, St. Louis, MO, USA) at 10 μM concentration for 0.5 hour.

### Microarray analysis

MiRNA microarrays were performed using Agilent's miRNA Microarray System (Agilent, Santa Clara, CA, USA) according to the manufacturer's instructions. Total RNA was quantified by the NanoDrop ND-2000 (Thermo Scientific) and the RNA integrity was assessed using Agilent Bioanalyzer 2100 (Agilent Technologies). The sample labeling, microarray hybridization and washing were performed based on the manufacturer's standard protocols. Briefly, total RNA were dephosphorylated, denaturated and then labeled with Cyanine-3-CTP. After purification the labeled RNAs were hybridized onto the microarray. After washing, the arrays were scanned with the Agilent Scanner G2505C (Agilent Technologies). Feature Extraction software (version10.7.1.1, Agilent Technologies) was used to analyze array images to get raw data. Next, Genespring software (version 13.1, Agilent Technologies) was employed to finish the basic analysis with the raw data. To begin with, the raw data was normalized with the quantile algorithm. The probes that at least 100.0 percent of samples in any 1 condition out of 2 conditions have flags in “Detected” were chosen for further data analysis. Differentially expressed miRNAs were then identified through fold change as well as *P value* calculated using *t-test*. The threshold set for up- and down-regulated genes was a fold change > = 2.0 and a *P value* < = 0.05.

### RNA isolation and qRT-PCR

Total RNA was isolated using Trizol reagent (Invitrogen, Carlsbad, CA, USA) according to the manufacturer's instructions. 1 μg of total RNA was reverse-transcribed to cDNA with PrimeScript^™^ RT Master Mix (Takara, Japan). qRT-PCR for target genes was performed on an Applied Biosystems 7300 Sequence. Detection System using SYBR green PCR Mix (iTAP, Bio-Rad). The reactions were incubated in a 96-well plate at 95°C for 10 min, followed by 40 cycles of 95°C for 15 s, 60°C for 30 s and 72°C for 30 s. The resulting amplification and melt curves were analyzed to ensure the identity of the specific PCR product. Threshold cycle values were used to calculate the fold change in the transcript levels by using the 2-ΔΔCT method. The relative mRNA expression levels were normalized to the β-actin gene. The method to quantify mature miRNAs was performed by stem-loop RT-PCR [43]using SYBR green PCR Mix (iTAP, Bio-Rad) with U6 snRNA as the internal reference control.1μg RNA and primers were put at 65°C for 5 min to form highly target-specific stem-loop structure, then reverse transcriptase, RNase inhibitor, dNTPs and 5 × buffer were added for reverse transcription. The amplification results were analyzed using 7300 System Software (V1.3.1, Applied Biosystems). The relative expression of each mRNA was calculated by the comparative ΔΔCt method [44]. The primer sequences are listed in Supplementary Table 1.

### Transient transfection

Hsa-miR-146a mimics, has-miR-146a inhibitor and negative control were double-stranded RNA oligonucleotides that were purchased from GenePharma (ShangHai, China). They were transfected into normal human THP-1 cells using Lipofectamine 2000 (Invitrogen) according to the manufacturer's instructions.

Three sequences of small interfering RNAs for Dectin-1 (si-dectin-1) were ordered from GenePharma. After transfection for 24 h, 48 h or 72 h, cells and the supernatants were harvested for the following experiments.

### ELISA assay

The expression of secreted IL-6 and TNFα in the supernatants of THP-1 cells with or without *Ca*IG after transfected with miRNAs oligonucleotides was detected using a specific commercial ELISA kit (Neobioscience, Shen Zhen, China) for IL-6 and TNFα according to the manufacturer's instructions.. After 48 h transfection, the cell supernatants were collected and centrifuged at 12000 × *g* for 10 mins, subsequently preserved at −80°C. All experiments were performed in triplicate (*n* = 3). The concentration of TNF-α and IL-6 was determined by measuring the absorbance at 450 nm with a microplate reader (MTP-32).

### Western blotting

After treatment, cells were washed two times with ice-cold phosphate-buffered saline (PBS) and then lysed in RIPA Lysis Buffer containing Protease Inhibitor Cocktail and the phosphatase inhibitor PhosSTOP (both from Roche Applied Science, Basel, Switzerland). The protein concentration of the whole cell lysate was quantified using BCA Protein Assay kit (Beyotime Biotechnology, Haimen, Jiangsu, China). Equal amounts of protein were separated by 12% SDS-PAGE (Beyotime Biotechnology) and were then transferred onto PVDF membranes (Millipore, Billerica, MA, USA). After blocking, the membranes were sequentially incubated with the indicated primary and secondary antibodies (Cell Signaling Technology). The protein bands were visualized using Page Ruler Plus Prestained Protein Ladder (Thermo Fisher Scientific, Waltham, MA, USA) using a chemiluminescence imaging method. The band intensities were quantified using Quantity One. β-Actin severed as the loading control.

### Nuclear translocation of NF-κB

The NF-κB-p65 in THP-1 cells was detected by indirect immunofluorescence assay using confocal microscopy. THP-1 cells were cultured on glass dishes. After the respective treatments, the cells were fixed using 4% paraformaldehyde, permeabilized with 0.2% Triton X-100 (w/v), and blocked with 3% bovine serum albumin (Sigma-Aldrich, Germany). Subsequently monoclonal antibodies against NF-κB-p65 (1:100) were applied for 12 h followed by 1 h incubation with anti-rabbit IgG conjugated to Texas Red antibody (1:400). Excitation and emission maxima used were 578 nm 602 nm respectively. The nuclei were visualized by staining with DAPI (excitation wavelength 359 nm and emission wavelength 461 nm).

### Flowcytomertry

100 μl cells were labeled with 10μl phycoerythrin (PE)-conjugated anti-human primate Dectin-1 monoclonal antibody (12-9856-41, eBioscience, San Diego, CA, USA) for 30 minutes at 4°C to determine the expression of Dectin-1. After washings, the cells were suspended in 200 μl PBS and analyzed by flowcytometry (BD FACS Verse^TM^, America). Cells were identified by the forward-scatter properties, and were subsequently included in a gate. Dectin-1 expression was assayed after different duration. The results were analyzed with the software Flow Jo 7.6.2.

### Luciferase reporter assay

A luciferase reporter plasmid pNF-κB-luc (1μg/ml) containing NF-κB response elements (Beyotime Biotechnology, Haimen, Jiangsu, China) was co-transfected into THP-1 cells with a renilla control plasmid (1μg/ml) and Pre-146a, Pre-NC (100 nM), Anti-146a or Anti-NC (200 nM) using Lipo2000 transfection reagent (Invitrogen, Carlsbad, CA, USA). 24 hours after transfection cells were treated with medium or *Ca*IG for 6 hours. Luciferase activity was analyzed using the Dual-Luciferase Reporter Gene Assay Kit (Beyotime Biotechnology).

### Statistical analysis

Results were presented as mean ± standard error of the mean (SEM). Graphpad prism version 5.01 was used to perform graphics and the Student's *t-test* and One-way analysis of variance were used to analyze the significance between groups. *P* < 0.05 was set as a statistical significance.

## SUPPLEMENTARY MATERIALS FIGURE AND TABLE


