# Supplementary material for: MiR-146a negatively regulates dectin-1-induced inflammatory responses
Source: Oncotarget. 2017 Apr 8;8(23):37355–66. doi: 10.18632/oncotarget.16958 (PMC5514914; doi:10.18632/oncotarget.16958)
Supplement: Supplementary file 1 [file oncotarget-08-37355-s001.pdf]

MiR-146a negatively regulates dectin-1-induced inflammatory responses

Supplementary Materials

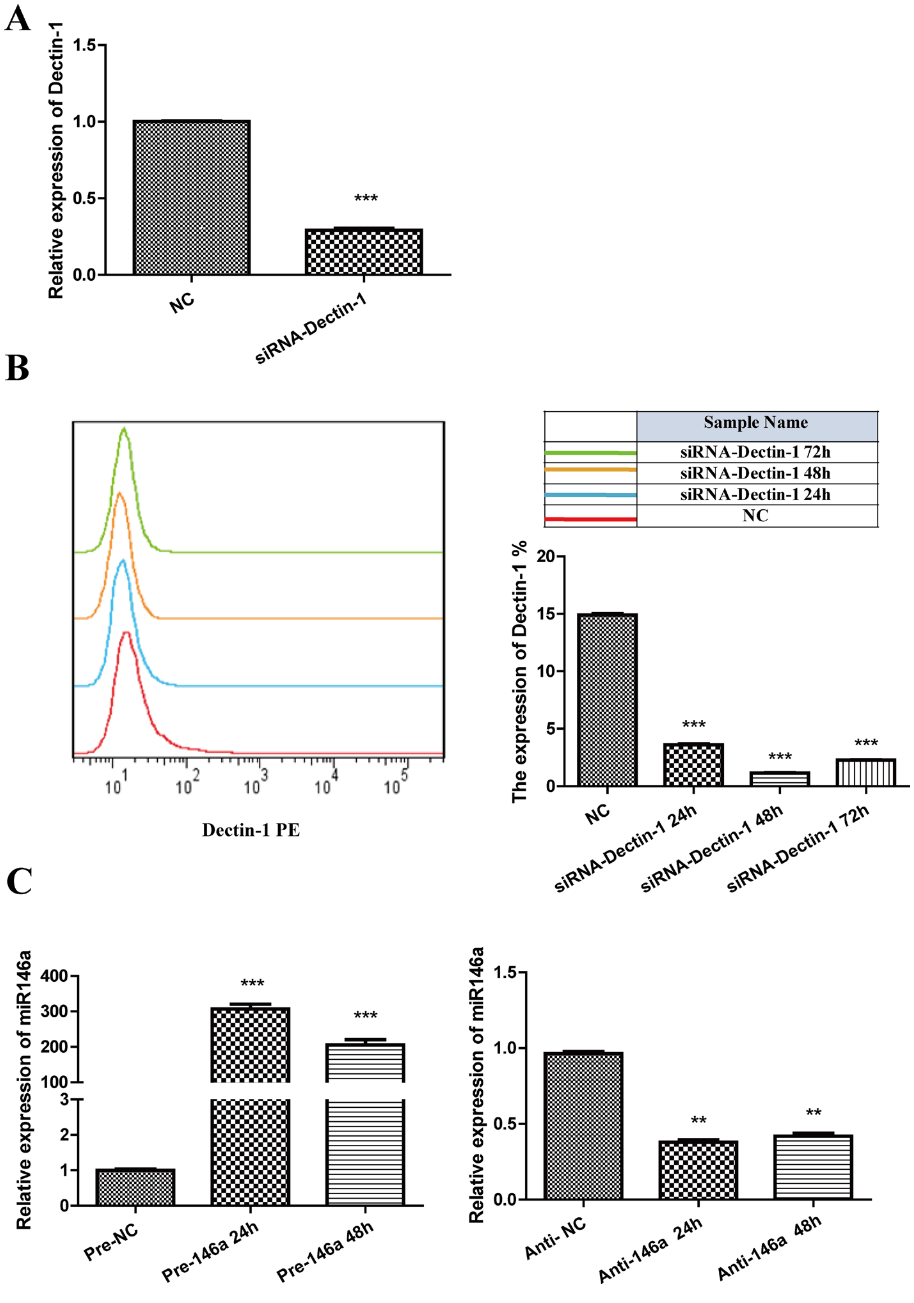

Supplementary Figure 1: Transfection efficiency of miRNA and siRNA. The (**A**) gene and (**B**) protein level of dectin-1 in THP-1 cells was respectively detected by qRT-PCR and flow cytometry in THP-1 cells transfected with dectin-1-specific siRNA (siRNA-dectin-1) or control siRNA (NC). (**C**)The gene level of miR-146a was detected by qRT-PCR normalized to U6 expression in THP-1 cells transfected with hsa-miR-146a mimics (Pre-146a), has-miR-146a inhibitor (Anti-146a) and negative control (Pre-NC and Anti-NC). Values are means ± S.E.M. from three experiments performed in triplicate. $^{**}P < 0.01$; $^{***}P < 0.001$.

Supplementary Table 1: Sequence information for qRT-PCR primers used in described studies

GENE_NAME	Forward Primer (5′-3′)	Reverse Primer (5′-3′)
β-actin	TCTGGCACCACACCTTCTA	AGGCATACAGGGACAGCAC
IL-6	TTCGGCAAATGTAGCATG	AATAGTGTCCTAACGCTCATAC
TNFα	CGAGTGACAAGCCTGTAGC	GGTGTGGGTGAGGAGCACAT
U6	CTCGCTTCGGCAGCACA	AACGCTTCACGAATTTGCGT
miR-146a	ACACTCCAGCTGGGTGA GAACTGAATTCCA	CTCAACTGGTGTCGTGGAGTC GGCAATTCAGTTGAGAACCCATG
miR-30a-5p	ACACTCCAGCTGGGTGTA AACATCCTCGAC	CTCAACTGGTGTCGTGGAGTCGG CAATTCAGTTGAGCTTCCAGT
miR-210-3p	ACACTCCAGCTGGGCTGTG CGTGTGACAGC	CTCAACTGGTGTCGTGGAGTCGG CAATTCAGTTGAGTCAGCCGC
miR-193a-5p	ACACTCCAGCTGGGTGGGT CTTTGCGGGCG	CTCAACTGGTGTCGTGGAGTCGG CAATTCAGTTGAGTCATCTCG
miR-5787	ACACTCCAGCTGGGGGGCT GGGGCGCGG	CTCAACTGGTGTCGTGGAGTCGGC AATTCAGTTGAGACCTCCCC
miR-8072	ACACTCCAGCTGGGGGCGGC GGGGAGGT	CTCAACTGGTGTCGTGGAGTCGGCA ATTCAGTTGAGCTGCCTAC
